# Impaired effective functional connectivity of the sensorimotor network in interictal episodic migraineurs without aura

**DOI:** 10.1186/s10194-020-01176-5

**Published:** 2020-09-14

**Authors:** Heng-Le Wei, Jing Chen, Yu-Chen Chen, Yu-Sheng Yu, Xi Guo, Gang-Ping Zhou, Qing-Qing Zhou, Zhen-Zhen He, Lian Yang, Xindao Yin, Junrong Li, Hong Zhang

**Affiliations:** 1 Department of Radiology, The Affiliated Jiangning Hospital with Nanjing Medical University, Nanjing, 211100 Jiangsu China; 2 Department of Neurology, The Affiliated Jiangning Hospital with Nanjing Medical University, Nanjing, 211100 Jiangsu China; 3 Department of Radiology, Nanjing First Hospital, Nanjing Medical University, No.68, Changle Road, Nanjing, 210006 Jiangsu Province China

**Keywords:** Magnetic resonance imaging, Migraine, Sensorimotor network, Effective functional connectivity

## Abstract

**Background:**

Resting-state functional magnetic resonance imaging (Rs-fMRI) has confirmed sensorimotor network (SMN) dysfunction in migraine without aura (MwoA). However, the underlying mechanisms of SMN effective functional connectivity in MwoA remain unclear. We aimed to explore the association between clinical characteristics and effective functional connectivity in SMN, in interictal patients who have MwoA.

**Methods:**

We used Rs-fMRI to acquire imaging data in 40 episodic patients with MwoA in the interictal phase and 34 healthy controls (HCs). Independent component analysis was used to profile the distribution of SMN and calculate the different SMN activity between the two groups. Subsequently, Granger causality analysis was used to analyze the effective functional connectivity between the SMN and other brain regions.

**Results:**

Compared to the HCs, MwoA patients showed higher activity in the bilateral postcentral gyri (PoCG), but lower activity in the left midcingulate cortex (MCC). Moreover, MwoA patients showed decreased effective functional connectivity from the SMN to left middle temporal gyrus, right putamen, left insula and bilateral precuneus, but increased effective functional connectivity to the right paracentral lobule. There was also significant effective functional connectivity from the primary visual cortex, right cuneus and right putamen to the SMN. In the interictal period, there was positive correlation between the activity of the right PoCG and the frequency of headache. The disease duration was positively correlated with abnormal effective functional connectivity from the left PoCG to right precuneus. In addition, the headache impact scores were negatively correlated with abnormal effective functional connectivity from the left MCC to right paracentral lobule, as well as from the right precuneus to left PoCG.

**Conclusions:**

These differential, resting-state functional activities of the SMN in episodic MwoA may contribute to the understanding of migraine-related intra- and internetwork imbalances associated with nociceptive regulation and chronification.

## Introduction

Migraine is an incapacitating neurological disorder, typically characterized by unilateral, throbbing or pulsating headaches, and the second-largest contributor to global neurological disorders, after stroke [1]. It affects more than 10% of the general population, thus causing enormous individual and social burden. Patients with migraine often experience anxiety, depression and sleep disturbances, which enhances the mental disorder and significantly impair the quality of daily life [2]. In order to explore effective treatment options for migraine headaches, it is imperative to understand the neuropathological mechanisms of migraine.

A migraine attack is not only a somatic perturbation, but also a sensory dysfunction that amplifies light, sound or touch perceptions in both ictal and interictal phases [3]. These characteristics make migraine a somatosensory disorder that eventually induce aberrant neuroplastic alterations in the central nervous system (CNS). In the last decades, neuroimaging has greatly contributed to the understanding of the neurological diseases involved in migraine pathophysiology. The neuroimaging data have shown that the disorder is associated with microstructural and functional alterations in various brain regions including the sensory motor network (SMN) [4]. The SMN, previously implicated in migraine, is a crucial region that is a multipurpose high-order cognitive processing center [5], which encompass the primary somatosensory cortex, primary motor cortex, premotor cortex and supplementary motor area [6]. Previous migraine resting-state functional magnetic resonance imaging (Rs-fMRI) studies have demonstrated functional alterations in some SMN subregions associated with pain and cognition [7, 8]. Besides, the migraine attack is a paroxysmal dysfunctional alteration or inflow-outflow dysfunctional modulation of multiple sensory systems [9]. Various neuroimaging outcomes have demonstrated correlation of functional connectivity between the subregions in SMN, and the central executive network (CEN) [6], salience network (SN) [10] as well as default mode network (DMN) [11] in patients with MwoA. The functional connectivity strength of SMN has been shown to significantly illuminate the perception of pain intensity and therapeutic effect in MwoA [12].

Whereas many studies have found that MwoA is associated with changes in functional connectivity between the SMN and other regions, it has been difficult to discern the directionality or specificity of the disrupted connections. Here, we selected the independent component analysis (ICA) method, a data-driven method, to separate the spatiotemporal component from the whole brain and extract the distribution of the SMN in a standard template. Using the Granger causality analysis (GCA), we identified differences in the direction of the SMN functional connectivity between patients with MwoA and healthy controls (HCs). Taken together, we observe that, akin to other pain disorders, migraineurs without aura would exhibit abnormal functional alterations within the SMN and the disruptions might define the nociceptive transmission pathway related to the SMN in the interictal resting state. Moreover, the disruptions of the SMN effective connectivity would be associated with specific migraine characteristics.

## Methods

### Participants

All episodic patients with MwoA were screened and classified following the criteria outlined by the third edition of the International Classification of Headache Disorders (ICHD-3; Code 1.1) [13]. By reviewing family medical history, we enrolled age- and gender-matched HCs, who were headache-free and whose family members did not suffer from any form of headache. We excluded patients who had brain organic disorders, other mental diseases, family history of mental retardation, dementia or physical disease, history of alcohol abuse, and any MRI contraindications. MwoA patients were headache-free and drug-free for at least 3 days prior to and after scanning to ensure that the patients were in the interictal period and avoid pharmacological interferences on signal fluctuation. For female participants, the data was recorded at mid-cycle to avoid hormonal influences on cortical excitement. Clinical characteristics such as age at onset, frequency of attacks, pain intensity (visual analogue scale (VAS)), and headache impact (Headache Impact Test (HIT)-6) were collected. Additionally, for all the participants, psychiatric assessments including Self-rating Anxiety Scale (SAS) and Self-rating Depression Scale (SDS), were also conducted to assess depression and anxiety state. This study was approved by the Medical Ethics Committee of Nanjing Medical University. Informed written consent was obtained from all the participants.

### Data acquisition

All data were acquired using a 3.0 Tesla MRI scanner (Ingenia, Philips Medical Systems, Netherlands) with an 8-channel head coil. Head motion and scanner noise were reduced by custom-fit foam pads and earplugs. The Rs-fMRI images were obtained axially using a gradient echo-planar imaging sequence as follows: repetition time (TR) 2000 ms, echo time (TE) 30 ms, flip angle (FA) 90°, number of slices 36, field of view (FOV) 220 × 220 mm^2^, matrix size 64 × 64, slice thickness 4 mm, and total 230 volumes. Structural images were acquired by a three-dimensional turbo fast echo (3D-TFE) sequence. The parameters were as follows: TR/TE 8.1/3.7 ms; slices 170; thickness 1 mm; gap 0 mm; FA 8°; acquisition matrix 256 × 256; FOV 256 mm × 256 mm. None of the subjects recorded any discomfort or fell asleep during scanning. No obvious structural damage was observed based on the conventional MR images.

### Data preprocessing

Using an automated preprocessing pipeline, we preprocessed the data using the Rs-fMRI Data Analysis Toolkit plus (RESTplus, http://restfmri.net/forum/). In the functional data pipeline, we discarded the first 10 time-point resting-state data due to the instability of the initial MRI signals. Then the remaining images were corrected for slice-timing and realigned to the middle volume using a six-parameter rigid body transformation. The generated images underwent spatial normalization into the Montreal Neurological Institute standard space at a resolution of a 3 × 3 × 3 mm^3^ and then smoothed using an isotropic Gaussian kernel (full width at half maximum = 8). After removal of the linear trend of the time courses, the temporal band-pass filtering (0.01–0.08 Hz) was performed to reduce the effects of low- and high-frequency physiological noise. Besides, to further reduce the effects of confounding factors unrelated to specific regional correlation, we used linear regression to remove several sources of spurious variance. The sources include six head motion parameters and average signals from cerebrospinal fluid (CSF) and white matter (WM). To avoid the risk of obtaining spurious negative correlations, we did not remove the global signal. Subjects with maximum head translation or maximum rotation that exceeded 2 mm or 2° respectively, were excluded.

Voxel-based morphometry (VBM) analysis was performed to segment the cerebral tissues into gray matter (GM), WM or CSF using the VBM8 toolbox (http://dbm.neuro.uni-jena.de/vbm), as previously described [14]. Subsequently, GM segments were modulated (with linear components) in order to preserve the absolute regional amount of GM from distortions. Finally, the modulated GM volumes were smoothed with a Gaussian kernel of 8 mm FWHM. We reported surviving clusters of voxels exceeding a voxel-level threshold of *p* <  0.001 (uncorrected) or a cluster size threshold of *p* <  0.05, family-wise error (FWE) correction for multiple comparisons.

### ICA processing

To investigate the independent components in MwoA and HCs, the smoothed data were analyzed using the Group ICA of fMRI Toolbox (GIFT, http://mialab.mrn.org/software/gift/). On the other hand, the preprocessed functional images for all the subjects were temporally concatenated to create a single four-dimensional data set, while the independent components were automatically estimated against consistent time-dependent functional activity. We then selected the SMN from these components as the best-fit component as previous studies [15, 16]. After estimating the group spatial maps, we performed back reconstruction of participant-specific spatial maps using the GICA method and then created output images for the components normalized with Fisher’s z-transformation. One-sample *t*-tests were utilized to extract principal component images per group with the FWE correction (*p* <  0.001). Subsequently, two-sample t-tests were conducted on the sensorimotor component with the false discovery rate (FDR) correction (*q* <  0.001). Age, sex, GM and education were used as covariates.

### GCA processing

We used GCA to detect the effective functional connectivity between the regions of interest (ROIs) defined above in SMN and the rest of whole brain. The GCA patterns were performed in the RESTplus software. GCA estimates the causal effects of the ROIs of SMN (X) on every other voxel in the brain (Y). A positive coefficient from X to Y indicates that activity in region X exerts a causal influence on the activity region Y; while, a negative coefficient from X to Y shows that the activity of region X exerts an adverse influence on the activity of region Y. The effective functional connectivity maps were created using the Pearson correlation coefficient between the reference time course by averaging the time series of each ROI and these time series with whole brain voxels. All Pearson correlation (*r*) values were then converted to z-scores by Fisher z-transformation. Further details and formulations of the GCA method can be found in previous studies [17, 18]. For group-level comparison, clusters passing a threshold of *p* < 0.001 (uncorrected) were deemed significant regions with the consideration of age, sex, GM and education as covariates.

### Correlation analysis

To investigate the association between abnormal activity or effective connectivity strength of the SMN and clinical characteristics, the mean ICA values within the significant ROIs and the Pearson correlation values were extracted and correlated with the clinical features using partial correlation analysis. Age, sex, GM and education were used as covariates. Statistical analyses were performed in SPSS 24.0 and the threshold was set at *p* < 0.05.

## Results

### Demographic and clinical information

Compared with the HCs, MwoA patients showed no significant differences in the age, sex, educational level or brain volumes, as shown in Table 1. However, migraineurs demonstrated higher scores in psychiatric tests than the HCs.

**Table 1:** Demographic, clinical and psychiatric characteristics of the participants

	MwoA	HCs	*p* value
Age (year)^a^	35.15 ± 10.18	35.50 ± 8.59	0.875
Sex (male/female)	6/34	5/29	0.758
Education (year) ^a^	12.15 ± 3.02	13.41 ± 3.16	0.084
Disease duration (year)^b^	5.5 (2,10.75)	/	/
Frequency (days/month) ^b^	3 (3,5)	/	/
HIT-6 score ^a^	58.45 ± 9.32	/	/
VAS score ^b^	6 (5,7)	/	/
SAS score ^b^	35 (29,47)	23 (22,26)	< 0.001
SDS score ^b^	35 (29,50)	24 (23,26)	< 0.001
Gray matter (mm^3^) ^a^	633.25 ± 56.72	619.48 ± 54.18	0.292
White matter (mm^3^) ^a^	492.47 ± 44.35	499.94 ± 55.22	0.521
Cerebrospinal fluid (mm^3^) ^a^	215.83 ± 19.42	217.90 ± 27.75	0.708
Brain parenchyma (mm^3^) ^a^	1125.72 ± 92.20	1119.42 ± 92.48	0.771

Data are presented as mean ± SD ^a^ or medians and interquartile ranges (25th–75th percentiles) ^b^

*HIT-6* Headache Impact Test-6, *VAS* visual analogue scale, *MwoA* migraine without aura, *HCs* healthy controls

### VBM findings

The current study did not reveal any significant structural differences between the two groups, neither at a statistical threshold corrected for multiple comparisons (FWE corrected, *p* < 0.05) nor at an uncorrected threshold (*p* < 0.001, cluster size > 50).

### ICA findings

**Fig. 1:**
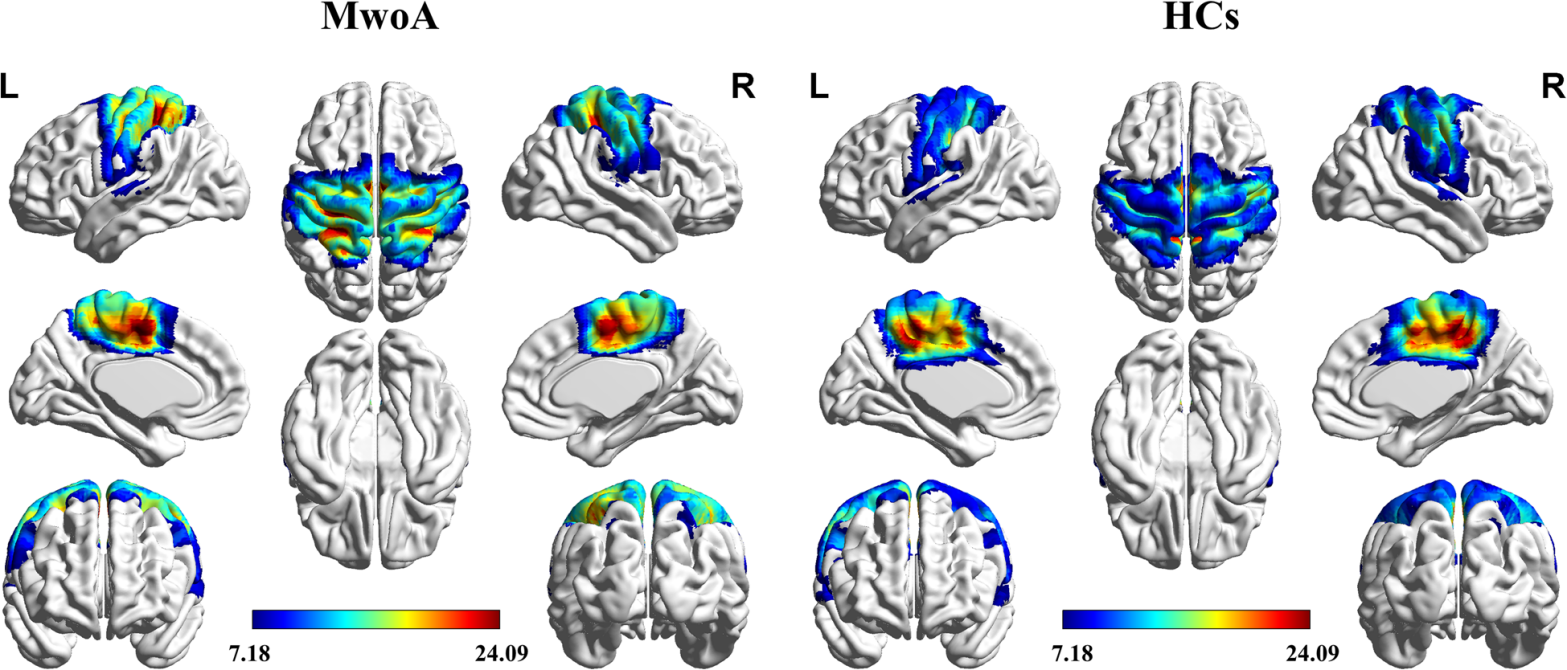
Group-level sensorimotor network in migraineurs without aura (MwoA) and healthy controls (HCs). Significant thresholds were corrected by family-wise error correction (*p* < 0.001)

The SMN for all the participants was extracted and reconstructed to group-level maps by ICA. The spatial positional distribution of the resting-state SMN is shown in Fig. 1. Inter-group comparison within the SMN revealed three significant ROIs: the bilateral postcentral gyri (PoCG) with increased activity, and the left midcingulate cortex (MCC) with decreased activity (Table 2).

**Table 2:** Significant differences in the SMN in patients with MwoA compared with HCs

Brain regions	X	Y	Z	K	T score
R postcentral gyrus	36	−15	69	20	9.7447
L postcentral gyrus	−27	−33	75	66	9.5054
L midcingulate cortex	−3	−39	45	515	−6.9314

*SMN* sensorimotor network, *MwoA* migraine without aura, *HCs* healthy controls, *L* left; *R* right

Significant thresholds were corrected by false discovery rate correction (*q* < 0.001)

### GCA findings

**Fig. 2:**
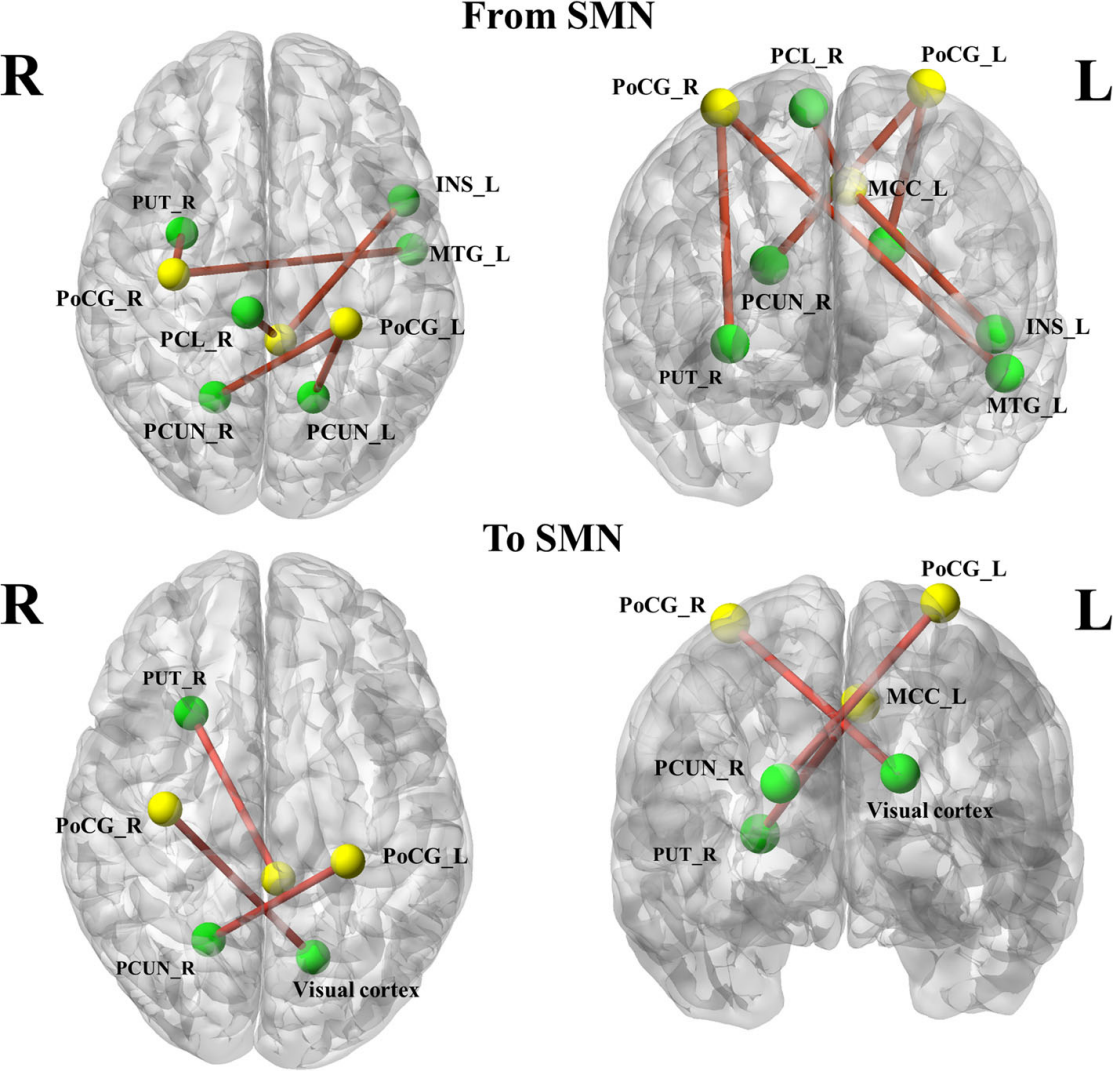
Altered effective connectivity from the SMN to other regions (First row) and from the other brain regions to SMN (Second row) in migraineurs without aura compared with healthy controls. Thresholds were set at a *p* < 0.001 (uncorrected). INS: insula; MCC: midcingulate cortex; MTG: middle temporal gyrus; PCL: paracentral lobule; PCUN: precuneus; PoCG: postcentral gyrus; PUT: putamen; SMN: sensorimotor network; L: left; R: right

Two-sample t-test findings of resting-state effective functional connectivity from the SMN to the rest of the brain are illustrated in Fig. 2 (first row, *p* < 0.001 uncorrected) and Table 3. Compared with the HCs, MwoA patients showed significantly decreased effective functional connectivity from the right PoCG to left middle temporal gyrus (MTG) and right putamen, and from the left PoCG to bilateral precuneus. On the other hand, the left MCC showed lower causal influence on the left insula and higher causal influence on the contralateral paracentral lobule (PCL).

**Table 3:** Altered effective connectivity between SMN and rest of brain in MwoA compared with healthy controls

			X	Y	Z	K	T score
From SMN							
R postcentral gyrus	→	L middle temporal gyrus	−51	−6	−12	13	−4.2655
		R putamen	33	0	−3	25	−4.1288
L postcentral gyrus	→	R precuneus	21	−60	21	63	−4.2511
		L precuneus	−15	−60	27	31	−3.9393
L midcingulate cortex	→	L insula	−48	12	0	10	−4.2464
		R paracentral lobule	9	−29	69	46	4.7412
To SMN							
R postcentral gyrus	←	primary visual cortex	−15	−66	24	206	4.3687
L postcentral gyrus	←	R precuneus	21	−60	21	19	4.3862
L midcingulate cortex	←	R putamen	27	18	6	40	4.4374

*MwoA* migraine without aura, *SMN* sensorimotor network, *L* left, *R* right

In addition, the two-sample t-test findings of resting-state effective functional connectivity from the whole brain to SMN are illustrated in Fig. 2 (second row, *p* < 0.001 uncorrected) and Table 3. Here, our data showed as significantly increased effective functional connectivity from the primary visual cortex (including the calcarine sulcus and cuneus) to right PoCG, and from the right precuneus to left PoCG. Moreover, the right putamen also showed increased effective functional connectivity to the left MCC.

### Correlation analysis results

**Fig. 3:**
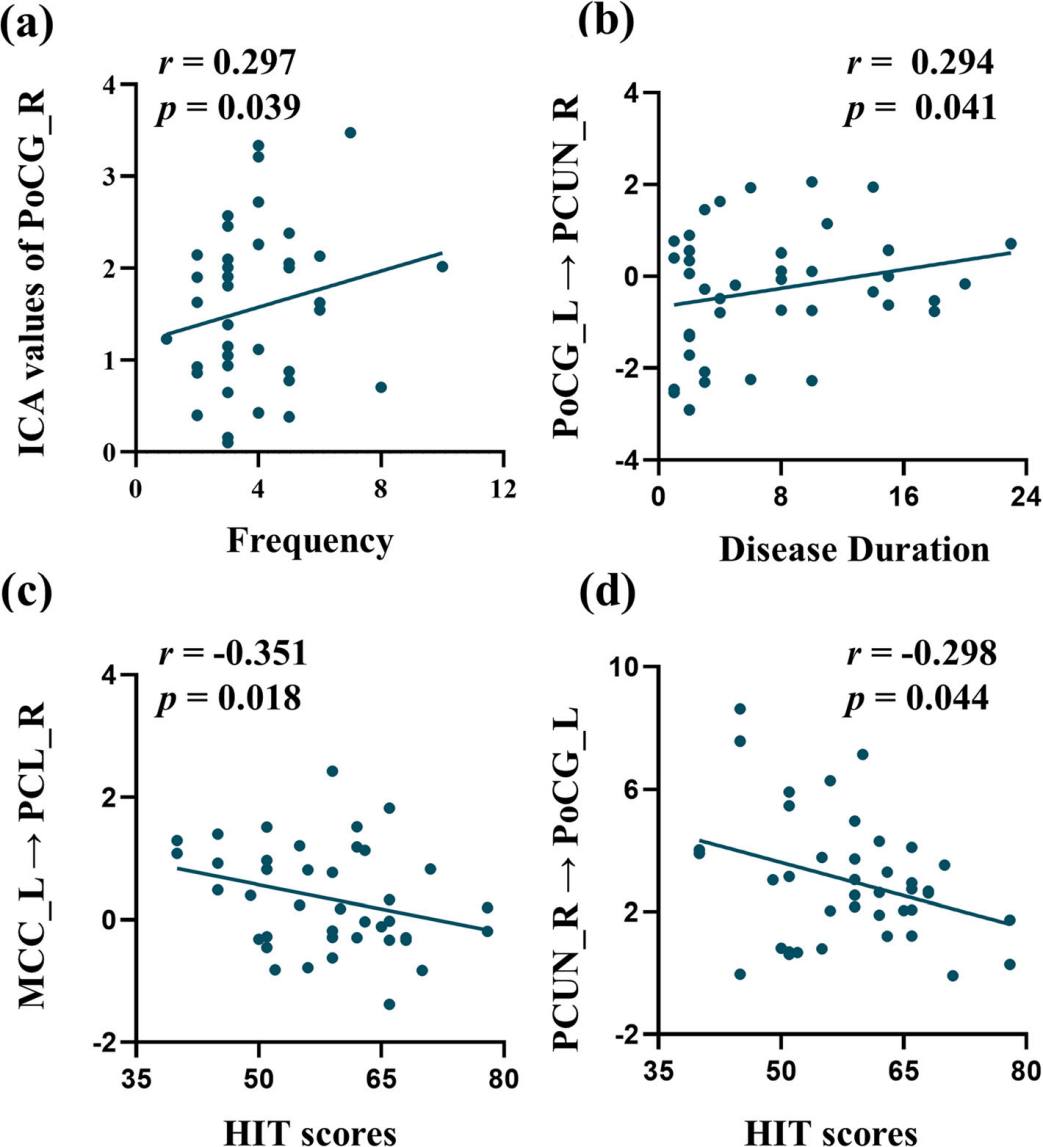
Significant correlations between clinical characteristics and abnormal connectivity in migraineurs without aura. HIT: headache impact test; ICA: independent component analysis; MCC: midcingulate cortex; PCL: paracentral lobule; PCUN: precuneus; PoCG: postcentral gyrus; L: left; R: right

The activity of regions in SMN significantly correlated with headache features, as shown in Fig. 3. There was positive correlation between the activity of the right PoCG and the frequency of headache (*r* = 0.297, *p* = 0.039). The disease duration was positively correlated with abnormal effective functional connectivity from the left PoCG to the right precuneus (*r* = 0.294, *p* = 0.041). In addition, the HIT scores were negatively correlated with abnormal effective functional connectivity from the left MCC to the right paracentral lobule (*r* = − 0.351, *p* = 0.018), as well as from the right precuneus to left PoCG (*r* = − 0.289, *p* = 0.044).

## Discussion

There are numerous challenges in discerning the directionality or specificity of the changes in functional connectivity between the SMN and other regions, especially in patients with MwoA. In this study, we extracted the distribution of SMN and identified abnormal neural activity of bilateral PoCG and left MCC unique to patients with MwoA. Specifically, the SMN in episodic patients with MwoA exhibited abnormal inflow or outflow influences on multiple functional networks and headache dysregulation related to clinical characteristics (e.g., headache frequency and duration). Our findings are in agreement with prior evidence of a disrupted SMN functional connectivity in migraineurs without aura [6], thus providing new insights into multi-sensory modulation in migraine processing. Moreover, we demonstrate that these effective functional abnormalities are independent of structural and microstructural changes.

In the current study, we found higher neural activity in bilateral PoCG, decreased effective functional connectivity from the right PoCG to the left MTG, as well as increased effective functional connectivity from the primary visual cortex (the calcarine sulcus and cuneus) to the right PoCG. The calcarine sulcus and cuneus are core regions of the primary visual network in the brain, and therefore, the present findings suggest that functional abnormalities between the SMN and visual network are specifically altered in patients with MwoA. Indeed, previous migraine studies have showed that altered activity within the sensory-related cortex, including SMN and visual cortex, results in dysfunction associated with affective, cognitive or pain processing [19, 20]. Moreover, previous study has shown altered function associated with nociceptive processing and cognitive impairment within the MTG in migraineurs [21]. Furthermore, a longitudinal investigation [22] showed that the morphological alteration of the visual cortex was significantly associated with migraine progression, especially in the calcarine sulcus and cuneus. Our findings also showed increased brain activity of the right PoCG, which is positively correlated with the headache frequency, and demonstrated functional deficits to compensate recurrent pain stimuli. Therefore, the abnormal effective functional connectivity between the SMN and visual cortex may be part of the pathological mechanism of failure to filter the unpleasant signals or lower the threshold to somatosensory stimuli in the visual pathway. Besides, the pain-induced task-state study demonstrated that episodic migraineurs had hyperactivation of the somatosensory cortex in the interictal phase [23]. These results are in line with the ones obtained from our analysis. However, Wang [8] and Zhao [24] et al. reported opposite resting-state neural activity of the bilateral PoCG in migraineurs with all headache subphases by low-frequency oscillations and regional homogeneity approaches to reflect the spontaneous neural function of the brain. In addition, an electroencephalography-related study [25] demonstrated higher desynchronization and power overlying the primary sensorimotor cortex in the preictal phase compared to the interictal phase, with no significant differences between interictal migraineurs and HCs. The heterogeneity of the migraineurs phase and the use of different neuroimaging methods might explain the discrepancy between the studies. Taken together, migraine is a chronic and periodic disorder of the central nervous system related to cortical and subcortical networks alterations of disrupting brain homeostasis and amplifying the intensity of sensory stimuli [26]. Further longitudinal investigation of how physiological changes within the migraine cycle is crucial to acquire a more complete understanding of the neuropathological mechanisms behind the headache attack.

Compared with the HCs, migraineurs without aura showed changed effective functional connectivity from the left PoCG to many pain-related areas, such as precuneus [5, 27]. This region has been demonstrated to be crucial in the DMN [28]. The DMN, one of the core brain networks that is activated when at a rest state, plays a pivotal role in discriminative, cognitive and perceptive functions of pain [20, 21, 29]. Moreover, the precuneus participates in the discrimination of sensory perception of pain [30] and the brainstem-thalamus-cortex circuit which modulates pain intensity [31] in the migraineurs. Our data found the increased brain functional connectivity from the right precuneus to left PoCG was negatively correlated with HIT scores, indicating that the dysfunction between the primary somatosensory cortex and DMN could disrupt the neural transmission pathway of regulation involved in sensory perception of pain. Whereas, the decreased brain functional connectivity from the left PoCG to right precuneus has a positive modulatory effect on the headache duration, suggesting that the long-term and repetitive migraine headache attacks could lead to somatosensory cortex compensatory or dysfunctional changes. These observations showed that influences between the left somatosensory cortex and DMN play a role in functional adaption along migraine progressing.

The current study also observed that, in resting state, there is decreased activity of the left MCC, a key region connected to circuits mediating cognitive control, nociceptive perception and multisensory integration [32, 33]. Particularly, it is suggested that the consecutive hyperactivation of the MCC could alleviate the neuropathic pain, but the hypoactivity of the MCC may diminish the pain inhibitory abilities, corresponding to the present findings [32]. This study provided evidence for functional changes from the left MCC to the left insula, which is localized near to the limbic system, subcortical network and anterior DMN, and may trigger pain processing adjustments in multiple instinct brain networks. The insula is a component of the SN (a pivotal large-scale intrinsic network associated with perceiving) related to the processing and integration of internal and external stimuli [34]. There are similar views on the contributions of MCC-associated networks spanning several cortical and subcortical networks to regulate the pain perception and processing. Notably, the pathway from the MCC to insula has been proven to be able to gate nociceptive hypersensitivity and amplify perception in the context of nociception [35]. Hence, the SMN-insula effective functional connectivity modulates the switching to the task processing. Since the SMN and insula are key regions of the trigeminovascular modulatory system, a pain inhibiting system, disrupted activity of these regions may lead to a dysfunctional pain inhibition pathway, thus contributing to the hypersensitivity of pain and migraine. Moreover, we found increased effective functional connectivity from the SMN to right PCL and negative correlation between the functional connectivity strength and HIT scores, similar to the results of previous Rs-fMRI study. Zhang et al. found weaker functional connectivity between the SMN and PCL in migraineurs without aura [6]. Thus, this pattern of the PCL dysfunction would induce abnormal sensorimotor integration, and as a possible abnormal neurophysiological mechanism in interictal MwoA.

In addition, one notable aspect revealed by this study is that the SMN subregions could be influenced by abnormal inputs from or outputs to the putamen, a component of the striatum. Literature has shown that the striatum affects the neuronal pathways underlying the inhibition effect of nociceptive stimulation [36]. These effects are mediated by the striatal dopamine D2 receptors which are associated with pain inhibitory circuitry of the caudal trigeminal nucleus [37]. The abnormal interaction of the putamen has been shown to trigger many independent components [38], justifying the hypothesis that transmission of pain is complex and multidimensional [39]. Since the cortico-striato-thalamo-cortical loop affects many neurological disorders [40], the results suggested that perturbation of the striato-cortical circuit may suppress the inhibitory function on the nociceptive reflex. Furthermore, the chronic pain and mood disorders have been proven to share common neuroanatomical substrates including the stratum [41]. The dysfunction of the striatum in chronic pain could disrupt the mesolimbic dopamine pathways and play a role in the pathogenesis of chronic pain and mood comorbidity [42]. Although no correlation between the brain dysfunction and the anxiety and depression scores was discovered in this study, MwoA patients showed higher measurement scores about anxiety and depression than HCs. Together with the previous evidence, we highlight the importance of putamen in pain-related processing as well as in the regulation of mood disorders comorbid with chronic pain syndromes.

Our study, however, used a small sample size. Therefore, a large sample size might be needed to enhance our data repeatability and reliability. In addition, the heterogeneity of the participants, such as the etiology, headache severity, disease duration, or neuropsychiatric comorbidity could result in neural activity biasness. Moreover, the pharmacological effects could play a pivotal role in the striato–thalamic–orbitofrontal pathway associated with pathogenesis of migraine and contribute to the abnormal brain function [37, 43]. The number of analgesic drugs consumed in a month should be carefully taken into account, meriting a more detailed study in the future. Besides the functional alterations, more studies are required to investigate the possibility of structural connectivity involved in SMN.

## Conclusions

In conclusion, we have explicated the abnormal interactions between the SMN and other networks in MwoA patients. Our data demonstrates that SMN plays a crucial role in pain modulation and chronification, as well as dissecting the neuropathologic mechanisms underlying episodic MwoA patients in headache-free phase.

## Data Availability

Clinical, neuroimaging and statistical data will be available upon request from any qualified investigator.

## Abbreviations

CSF: Cerebrospinal fluid
GM: Gray matter
HCs: Healthy controls
HIT: Headache impact test
ICA: Independent component analysis
MCC: Midcingulate cortex
MTG: Middle temporal gyrus
MwoA: Migraine without aura
PCL: Paracentral lobule
PCUN: Precuneus
PoCG: Postcentral gyrus
PUT: Putamen
Rs-fMRI: Resting-state functional magnetic resonance imaging
SAS: Self-rating Anxiety Scale
SDS: Self-rating Depression Scale
SMN: Sensorimotor network
VAS: Visual analogic scale
WM: White matter
